# The Influence of Osmotic Treatment, Edible Coatings Application, and Reduced Pressure on Microwave–Vacuum-Dried Carrot Properties

**DOI:** 10.3390/molecules30091877

**Published:** 2025-04-23

**Authors:** Anna Ignaczak, Łukasz Woźniak, Agata Marzec, Jolanta Kowalska, Małgorzata Chobot, Hanna Kowalska

**Affiliations:** 1 Department of Food Engineering and Process Management, Institute of Food Sciences, Warsaw University of Life Sciences, 159c Nowoursynowska St., 02-776 Warsaw, Poland; agata_marzec@sggw.edu.pl (A.M.); jolanta_kowalska@sggw.edu.pl (J.K.); malgorzata_chobot@sggw.edu.pl (M.C.); 2 Department of Fruit and Vegetable Product Technology, Institute of Agricultural and Food Biotechnology, State Research Institute, 36 Rakowiecka St., 02-532 Warsaw, Poland; 3 Department of Food Safety and Chemical Analysis, Institute of Agricultural and Food Biotechnology, State Research Institute, 36 Rakowiecka St., 02-532 Warsaw, Poland; lukasz.wozniak@ibprs.pl

**Keywords:** dried carrot snacks, osmotic enrichment, osmotic dehydration, edible coatings, phenolic content, antioxidant activity

## Abstract

The study investigated the effect of osmotic treatment, edible coatings, and reduced pressure on the quality of carrots dried by the microwave–vacuum method (MVD) at 3.5 or 6.5 kPa and microwave power of 250 W. Initial osmotic enrichment (OE) of carrots was carried out in chokeberry NFC juice, and osmotic dehydration (OD) in chokeberry juice concentrate. Coatings were prepared using sodium alginate or citrus pectin solutions of 1.0 or 1.5%. Osmotic treatment, and then drying pressure, had the greatest effect on increasing the dry matter (DM), total phenolic content (TPC), and color changes, but also on decreasing the water activity (AW) of dried carrot. The highest DM (average 98.7%) and the lowest AW (average 0.25) were obtained in OE carrots and dried at 3.5 kPa. Drying carrots, combined with osmotic treatment and coating, increased TPC by 13-fold, from 225 in fresh to 3229 mg GAE/100 g d.m. in dried carrots. Osmotic treatment did not affect the antioxidant activity of DPPH•, but OD significantly increased ABTS•+ compared to the raw material. Coatings had a smaller effect on color changes and antioxidant activity (DPPH• and ABTS•+) and no significant impact on DM and AW. The color changes of the control and coated samples were an increase in color lightness, redness, yellowness, and saturation (vividness), and those subjected to osmotic treatment showed a decrease in these parameters. The lower AW of dried carrots positively affected higher hardness. All samples were sensory accepted, including color, texture, and smell, especially after OD in chokeberry juice concentrate, while crunchiness was the lowest (five out of nine points).

## 1. Introduction

The challenge for food producers today is to provide consumers with safety and health benefits by limiting additives and generating a simple nutritional composition with increased natural or minimally processed ingredients. Simultaneously, production methods, environmental issues, and sustainability are becoming increasingly important [1]. Snacks occupy a large share of the food market. This wide range of products has gained popularity due to their convenience. However, they often contain many food additives, such as preservatives and compounds whose primary purposes are durability and shaping the desired sensory properties. Dried fruit and vegetable chips offer an interesting alternative as they are a source of many micro- and macro-elements, especially dietary fiber, and they also do not contain gluten or fatty acids with an unfavorable profile [2,3,4]. Consumers expect them to meet their appearance and taste expectations when choosing “healthy” snacks. Therefore, it is essential to maintain a balance between health aspects and sensory quality [5].

Dried carrots can be classified as “healthy” snacks. Carrot snacks can benefit the diet or satisfy hunger between main meals. Many studies have shown that carrots in various forms, including dried ones, contain high values of ingredients desirable in the human diet [3]. Carrots are a good source of precursors of vitamin A, vitamin C, and the B vitamin complex (B1—thiamine, B6—pyridoxine, B9—folic acid), as well as fiber, including pectin, which lowers cholesterol levels, and minerals including calcium, copper, magnesium, potassium, phosphorus, and iron [6,7,8]. In the study by Koca Bozalan and Karadeniz [9], the most important carotenoids in carrots were β-carotene (41.60–71.2 mg/kg fresh weight (FW)) and α-carotene (13.44–30.11 mg/kg FW), and the total phenolic content was 114–306 mg catechin/kg. In the review article by Sharma et al. [10], the protein content of carrots was listed in the range of 0.7–0.9%, fat as 0.2–0.5%, carbohydrates as 6.0–10.6%, fiber as 1.2–2.4%, and total ash as 1.1%, including Ca 34–80 mg/100 g, Fe 0.4–2.2 mg/100 g, P 25–53 mg/100 g), Na (40 mg/100 g), K (240 mg/100 g), Mg (9 mg/100 g), Cu (0.02 mg/100 g), Zn (0.2 mg/100 g), carotenes (5.33 mg/100 g), thiamine (0.04 mg/100 g), riboflavin (0.02 mg/100 g), niacin (0.2 mg/100 g), vitamin C (4 mg/100 g), and energy value (126 kJ/100 g). These ingredients make carrots, or their addition in various forms, such as raw, juice, puree, and pomace, an important component of the diet, essential for the functioning of the human body, among others, thanks to their nutritional and antioxidant effects, conditioning the growth and repair of tissues, improving eyesight, and helping to fight infections [2,3,9].

Various drying methods allow water activity to be reduced to a level low enough to inhibit the growth of microorganisms, enzymatic reactions, and other undesirable reactions that may occur during storage [11]. Microwave–vacuum drying (MVD) is particularly useful for producing high-quality dried products [12]. Short process times and reduced oxidation reactions affect the preservation of many natural ingredients and sensory qualities [3,13,14,15,16]. Many studies have been conducted to produce carrots by drying them using microwaves, and the microwave–vacuum method has proven particularly useful [3,6,15,16,17,18,19,20]. In the case of MVD of carrots or other raw materials, a preliminary reduction of the water content in the raw material is required [15,20,21]. In addition to reducing the moisture content of carrots, convective pre-drying creates a crust around the product, which increases the puffing effect due to higher pressures inside the product caused by water vapor [15,22]. Pre-treatment is intended to improve the efficiency of the process and shape the properties of dried products. The appropriate selection of these methods influences sensory values and allows for limiting the loss of natural nutrients and health-promoting components or increasing them through enrichment.

Blanching is a necessary pre-treatment before drying carrots, among other things, to soften the texture and increase the drying speed [23]. The primary purpose of blanching is to inactivate naturally occurring enzymes [24], as well as to remove gases from the surface and intercellular spaces to prevent oxidation, discoloration, and development of unpleasant tastes and to reduce the initial number of microorganisms [4]. The presence of enzymes in unblanched carrots may result in low β-carotene content [25].

Osmotic dehydration (OD) is a commonly used treatment for partial moisture reduction before the drying of plant materials [26,27], and the use of osmotic pre-dehydration or convective drying is beneficial and required before microwave–vacuum drying when the raw materials contain high moisture [17,21]. According to Changrue and Orsat [17], osmotic dehydration at 50% sugar and 5% salt concentration for 158 min before this drying removed about 50% of the free water from carrots. In addition, OD was able to shorten drying time and energy consumption, reduce shrinkage, and improve the appearance of the product [17]. Currently, to reduce simple sugars in the human diet and shape the health-promoting properties of dried products, polyols [28] and fruit or vegetable juice concentrates are used as osmotic substances, which are a source of many valuable ingredients [21,29,30]. The use of non-concentrated NFC (not from concentrate) juices is mainly aimed at enriching plant tissue with natural juice ingredients, improving the sensory quality of the dried product and its health-promoting properties [4,20].

Applying edible coatings or films to post-harvest raw materials and other stages of food processing is a common procedure observed in many scientific studies and the food industry. The coatings are intended to preserve freshness, prevent changes in sensory properties, and protect against the microbiological contamination of fresh fruits and vegetables, thereby extending their shelf life. In addition, the coating protects the product from oxygen and changes in taste and aroma, including minimally processed carrots [31,32,33,34]. In a review article, Khalid et al. [35] demonstrated the effect of edible coatings on increasing the safety and quality of products. They drew attention to the use of coatings in response to consumer needs for chemical-free products with excellent quality and nutritional profiles. Many studies have used coatings before drying fruits or vegetables [19,36]. An appropriate selection of coating agents and manufacturing techniques can benefit the properties of foods, including dried foods, by minimizing color degradation and textural changes, potentially reducing shrinkage and ensuring the product’s aesthetic appearance [11]. Pectin is a valuable biomaterial for producing environmentally friendly edible coatings used in various stages of food processing and packaging of various food products [34]. Barrera-Chamorro et al. [34] indicated that pectins are characterized by technical, functional, and biological gel-forming and thickening properties and can contribute to microbiota modulation in animals and humans. They are suitable for a wide range of applications, such as packaging, carriers, or functional ingredients as a source of fiber. Alginate, a naturally occurring polysaccharide, acts as a humectant moisture agent, limiting physiological processes and improving mechanical properties; edible alginate coatings and films improve or maintain the quality and extend the shelf life of various types of food, including vegetables [37,38]. Mina et al. [39] indicated that compared to control samples, pre-coating carrot slices with 3% gum Arabic had a more beneficial effect on carrot color, water activity, total phenolic content, β-carotene, and DPPH• radical scavenging activity. In the literature, it is difficult to find the use of coatings before osmotic dehydration and even more difficult to find vegetables that are enriched before drying [40]. After osmotic treatment, their application to carrot slices aims to preserve the natural and enriching ingredients in dried carrots and improve their sensory quality.

The designated research objective was to determine the effect of edible coatings on the efficiency of enriching NFC juice ingredients and the quality of carrots produced by microwave–vacuum drying, depending on the value of reduced pressure. The direction of this research was to explain the mechanisms from the scientific side and, in the practical aspect, to improve the technology of producing dried carrots to increase their nutritional, health-promoting, and sensory attractiveness. These features encourage consumers to eat vegetables, which, in such a version, will be very beneficial for their health.

## 2. Results

### 2.1. Influence of Osmotic Treatment, Edible Coating, and MVD Pressure on Physical Properties of Dried Carrot

#### 2.1.1. Dry Matter Content (DM) and Water Activity (AW)

The state and content of water in food affect its properties related to structure, appearance, and shape, which simultaneously shape sensory features and nutritional value. Moisture is also a determinant of the durability and suitability of food for consumption. Higher water content in a product increases its susceptibility to the growth of microorganisms that can cause spoilage and negatively affect the texture of such dried products, making them rubbery instead of crispy and brittle. The most important indicators helpful in assessing changes in moisture content in dried products are dry matter content (DM) and water activity (AW). A negative correlation between dry matter content and water activity is typical in dried products (Table 1).

The purpose of drying vegetables is to obtain a low water content, which also means a high dry matter content. Depending on the production method of the dried product, the type of raw material, and the desired properties of the dried products, the moisture content in dried vegetables may vary. In the case of thin carrot slices with an initial thickness of approx. 3 mm, which should be brittle and breakable, the moisture content should be as low as possible, in the order of a few percent. For reasons of microbiological safety, water activity should not exceed 0.6 [41] and even 0.3 [12].

A significant effect of osmotic pre-treatment of carrots (enrichment (OE) in NFC chokeberry juice and osmotic dehydration (OD) in chokeberry juice concentrate) and drying pressure (3.5 and 6.5 kPa) on the dry matter content (DM) and water activity (AW) was found, but no significant effect of coatings on DM and AW was observed (Table 1).

Among the enriched samples, including those with pectin (P) and alginate (SA) coatings and dried at a pressure of 3.5 kPa, the dry matter content was in the range of 97.2–99.5% and reached the highest value in the case of the enriched samples without coatings (MVD_OE_3.5). The water activity for these samples was at the level of 0.24–0.27. Compared to OE, the initially osmotically dehydrated samples, except for one case, i.e., osmotically dehydrated and pectin-coated samples (MVD_3.5_OD_P1.5) with water activity of 0.16, showed slightly higher AW values in the range of 0.26–0.29. Despite the significant difference in concentration between the juice and the chokeberry concentrate, osmotic treatment did not cause any difficulties removing moisture from the samples during drying. This was probably due to the relatively short time of osmotic treatment and subsequent drying by the MVD method. However, the enrichment of thin (3 mm) carrot half-slices in a lower juice concentration facilitated drying, giving higher dry matter content values and lower water activities than osmotically dehydrated samples in chokeberry juice concentrate.

The significant effect of reduced pressure during microwave–vacuum drying resulted in the exclusion of drying carrots at a pressure of 6.5 kPa. In these conditions, the DM values were the lowest (84.6–93.6%), and AW (0.43–0.58) was significantly higher than 3.5 kPa. Moreover, despite the water activity being below 0.6, some samples could be insufficiently dry, with some samples being locally burnt. The higher pressure, being closer to atmospheric pressure, could have increased the boiling point of the water in the carrots and been less beneficial.

The increasing use of edible coatings and films is observed in many scientific studies and in the food industry. Coatings limit the physicochemical and sensory changes in food, protect against microbiological contamination, and thus extend the shelf life [34,41]. In this study, the coatings were designed to to limit losses of native and introduced compounds compounds during osmotic treatment. It can be assessed that the coatings in our studies did not fulfill these functions.

#### 2.1.2. Color Parameters and Absolute Color Difference (ΔE)

The color of a food product is an important quality indicator of dried products, which determines the consumer’s acceptance of the product, and is also an indicator of food quality control [12]. The evaluation of parameters using the CIE L*a*b* system provides many possibilities for characterizing the color of carrot samples in an objective manner that can be expressed in numerical values. The lightness, redness, and yellowness values indicate carrot color and quality, with higher values often indicating better color and quality [42]. The mean values of the L*, a*, b* parameters of crushed fresh carrots were 40.24, 15.28, and 26.53, respectively, with a standard deviation from 0.09 to 0.21 (Table 2), while in previous studies [4] they were 65.37 (L*), 25.58 (a*), and 36.83 (b*), respectively, and in the study by Doymaz [43], 60.04, 20.41, and 26.97. Crushing the samples before color measurement probably allowed for the reduction of the standard deviations, and the different values of the color parameters of fresh carrots could result from the cultivation and storage conditions. Mina et al. [39] showed that dried carrot slices pretreated with 3% gum Arabic showed higher yellowness, 25.82–34.50, and the total color difference was 8.12–13.06. According to Wang et al. [42], carotenoids responsible for the red and yellow colors in fresh carrots occurred in chromoplasts. After blanching, which destroyed the cell structure, they could penetrate the crystalline chromoplasts in the intercellular space and increase the share of red and yellow colors.

The color values (L*, a*, and b*) of dried carrots were compared with the color of fresh carrots, absolute color differences ΔE were calculated, and statistical analysis was performed (Table 2). According to the evaluation criteria by Wang et al. [42], the absolute color difference ΔE of all dried carrot samples corresponds to the color perception of the human eye. It can be perceived even by inexperienced observers.

A significant effect of coating (type and concentration) and osmotic treatment (enriching and dehydration), and no drying pressure effect on color ΔE changes were demonstrated. Compared with the color of the raw material, the color of all dried carrot samples differed significantly, and ΔE values were in the range of 7.96–35.78 (Table 2). The color changes of carrot samples without osmotic treatment and coatings consisted of significant lightening and reduction of the share of red color, especially in the case of samples dried at a pressure of 6.5 kPa, in which the share of yellow color was also reduced.

Similarly, the study by Keser et al. [44] showed a significant lightening of microwave-dried carrot samples (L* in the range of 63.4–74.3), with a higher microwave power resulting in smaller differences in color lightness compared to the color of the raw material (49.8). According to Amin et al. [45], the reduction in yellowness may be due to the formation of brown pigment due to non-enzymatic Maillard browning and degradation of the carotenoid pigment, especially in carrots dried at a higher microwave power (510 W) where moisture loss was rapid, compared to drying at 170 and 340 W where the color change was gradual. However, according to Calín-Sánchez et al. [12], rapid moisture removal to 15–20% can shorten the Maillard reaction time and limit color changes. Using 3% gum Arabic coating as one of the pre-treatment methods for carrots before ultrasonication and subsequent drying, Mina et al. [39] found that carrot slices exhibited a better yellowness color of 25.82–34.50 and a total color difference of 8.12–13.06 than the control or ethanol-dipped samples.

**Figure 1:**
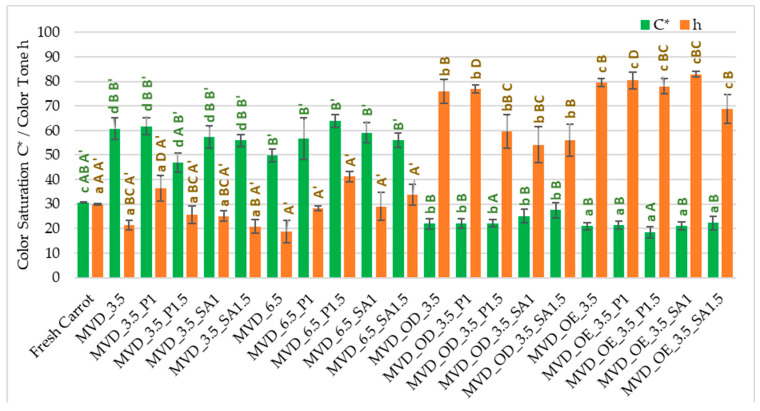
Color saturation C* and tone h parameters of dried carrots. Different letters mean homogeneous groups at *p* ≤ 0.05: a, b, c, d—osmotic treatment effect (enriching (OE)/dehydration (OD)) in NFC chokeberry juice/concentrate; A, B, C, D—coating type and concentration; and A’, B’—drying pressure. Code designations of dried samples are in Table S1.

The highest changes in ΔE (26.2–35.8) resulted from the darkening of the color (L*) and reduction of the share of red (a*) and yellow (b*) in the case of osmotically treated and coated samples. In the case of samples that were coated only, regardless of the type of coating, the color changes were also significant. They resulted from significantly higher values of all color parameters: color brightness L* by 1–4%, and especially redness, up to 1.5 times, and yellowness, up to 86% higher than in the color of fresh carrots. The absolute color difference in most of these samples was 17.6–29.3; only samples coated with pectin (1.5%) showed the smallest color changes ΔE, with a value of 8.0. The use of coatings without osmotic treatment resulted in the values of the color saturation index C* increasing by two times, which is an important marker of the intensity of the product’s color retained by the human eye [12], but with minor changes in the color tone h (Figure 1). On the other hand, in samples subjected to osmotic and enrichment treatment in chokeberry juice and juice concentrate, the opposite was the case. The C* parameter value was several percent lower, and the color shade was 1.8 times stronger than the color of fresh carrots. Using chokeberry juice and concentrate with a characteristic color for the initial processing of carrots had the most significant impact on the formation of the color of dried samples. It is also crucial in shaping the sensory values and could partially mask the uneven color of dried carrots. According to de Mendonça et al. [46], drying carrots using the microwave–vacuum method was significantly more beneficial than the convection method. An additional advantage was drying at a lower microwave power, which resulted in a lighter and more vivid color (C* saturation) and yellowness (color tone approx. 90°) of the samples. This was due to the lower drying temperature and less oxygen, preventing the formation of a darker or brown color. Michalska et al. [47] showed that the darkening of carrots during drying may be caused by oxidation reactions of carotenoids, especially β-carotene and Maillard, especially at high temperatures and in the presence of oxygen.

#### 2.1.3. Textural Properties

**Figure 2:**
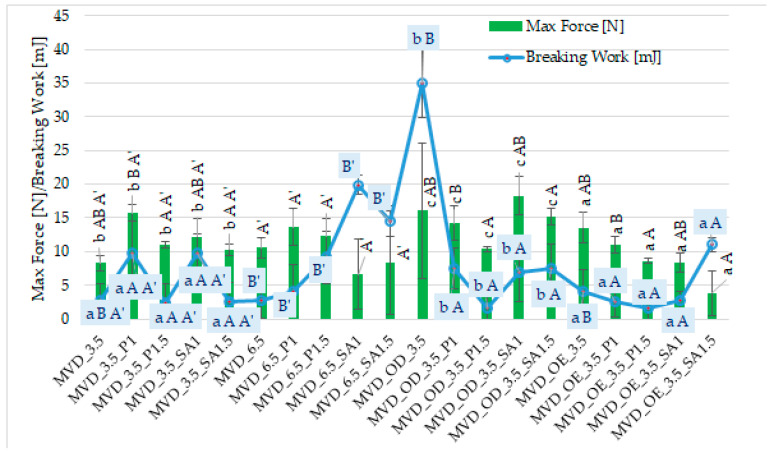
Mechanical properties of dried carrots. Different letters mean homogeneous groups at *p* ≤ 0.05: a, b, c—osmotic treatment effect (enriching (OE)/dehydration (OD)) in NFC chokeberry juice/concentrate; A, B—coating type and concentration; and A’, B’—drying pressure. Code designations of dried samples are in Table S1.

Among many food drying methods, microwave–vacuum drying is valued due to the relatively short drying time and high quality of dried products [13,14,15,48]. It was observed that dried carrot slices showed different values of maximum force (3.9–18.3 N) and breaking work (1.6–35.1 mJ) (Figure 2).

However, similar to the previous publication by Ignaczak et al. [20], the uneven shape of the dried samples caused large standard deviations of both indicators. Moreover, the values of these indicators in the current studies of 3 mm thick half-slice samples were significantly lower, and the breaking work was even several times lower than in previous studies on 5 mm whole slices [20]. This may be mainly due to the different sizes of the carrot samples, cultivation and storage conditions, and other properties of the dried products. Compared to previous studies, carrot samples dried by the microwave–vacuum method, including osmotic treatment and coatings, were more brittle but less crunchy. This was manifested by a smaller area under a much less “jagged” curve of the dependence of the breaking force on time. Statistical analysis showed a significant effect of osmotic treatment (enriching (OE)/dehydration (OD)) in NFC chokeberry juice/concentrate juice, coating type and concentration of coating solution, and also MVD pressure on the value of the breaking work, but no effect of pressure on the maximum breaking force of dried carrot samples (Figure 2).

In terms of the hardness of dried carrots (max force), osmotic treatment in the form of osmotic dehydration (OD) resulted in a higher hardness of the samples compared to the enriched samples (OE). Probably this was due to the greater effect of the initial osmotic dehydration of carrots, which was carried out in concentrated chokeberry juice with a concentration of approx. 67 ± 1 °Bx than during enrichment in NFC chokeberry juice with a much lower concentration of approx. 19 ± 1 °Bx. A higher concentration of osmotic solution increases the penetration of osmotic solution components into the dehydrated carrots, especially in the surface layers. This could have made it difficult to remove moisture during MVD. Hence, the lower dry matter content (Table 1) and the solution components could have increased the hardness of the carrot samples. Therefore, the breaking work was significantly higher compared to the other samples.

Despite the significant effect of the coating type on the texture properties of carrot samples, it is difficult to determine specific relationships. In most samples with coatings, higher values of both indices were observed than in the control samples without coatings. The average values of breaking work and maximum breaking force of the samples were the highest for samples with coatings without osmotic treatment (6.1 mJ and 12.3 N). OD samples with coatings showed a lower average breaking work (5.8 mJ) but a significantly higher maximum force (14.5 N) and lower values in OE samples with coatings (4.5 mJ and 7.9 N). In most cases, samples with pectin and alginate coatings produced from a 1% solution gave higher values of both indices than when using a 1.5% coating solution.

Changrue and Orsat [17], using osmotic treatment of 10 mm carrot cubes before MVD, found that the intake of osmotic substance from a mixed osmotic solution (50% sugar and 5% salt concentration) resulted in a softer product than that obtained without osmotic treatment. Due to the different types of cutting (shredding) of carrot samples and the lack of consideration of the fact that osmotic dehydration causes the penetration of osmotic substances mainly into the outer layers, it was not easy to compare these studies with our half-slices with an initial thickness of 3 mm. However, this confirms the beneficial use of osmotic treatment before MVD to obtain high quality dried fruit or vegetables in a short time [4,17,21,30]. This was associated with the necessary reduction of moisture content of OD samples before MVD and the increase in dry matter content, including through the penetration of osmotic substances. It was not easy to find similar studies using coatings for samples after osmotic treatment and drying using the MVD method. Nevertheless, based on the obtained data, it was shown that, in contrast to the alginate coating, in most samples, the pectin coating applied to carrot samples after their osmotic treatment (OD/OE) caused lower values of the breaking work, but higher values of the max. force in OE samples. Dried carrot slices coated with 3% gum Arabic were about three times harder than the control in the Mina et al. [39] study. Gumminess and hardness, which may change depending on humidity (water activity), are also associated with breaking/brittleness, which is desirable for snacks in the form of chips.

It was observed that the samples with the coating and without OD were slightly “rubberier”. As a result, during the breaking test, they did not always break after reaching the set deformation distance. Carrots that were osmotically dehydrated without the coating and dried at a pressure of 3.5 kPa, and then with the alginate coating, without OD and dried at a pressure of 6.5 kPa, were characterized by the highest breaking work, which could result from the greater rubberiness of these samples, giving a larger area under the breaking curve. Puffing, characteristic of and desirable when using the MVD method, can occur in some individual slices (chips). This can result from uneven drying (exposure to microwaves). Therefore, to exclude individual under-drying of chips, it seems reasonable to dry the entire batch of material slightly longer without using microwaves. Compared to microwave drying of carrots at atmospheric pressure [18], microwave–vacuum drying is recommended, resulting in lower density and less gumminess. De Mendonça et al. [46] presented the number of peaks under the strain curve to evaluate the crispness of dried carrot samples; the greater the number of peaks, the greater the crispness. They also found that increasing the microwave power reduced the hardness and increased the crispness of dried carrots using microwaves and reduced pressure.

#### 2.1.4. Structure (Scanning Electron Microscopy (SEM)) Analysis

Microwave–vacuum drying has a beneficial effect on the physicochemical properties of carrots [4,20]. These changes are related to the structure changes observed in the photographs shown in Table 3 and Table S2. Wang et al. [23] used a scanning electron microscope to study the structure of blanched carrots, and energy-dispersive X-ray spectroscopy observations allowed us to observe the mechanisms of texture softening and drying rate increase driven by blanching. Ciurzyńska et al. [49] proved that convectively and microwave-dried carrot tissue shows a regular distribution of pores in high-density clusters. However, numerous cell damages and cracks are visible. At the same time, freeze-dried samples are distinguished by many small-diameter pores, creating a delicate porous structure, which they explained is caused by the lack of high temperatures.

The effect of the applied pressure (3.5 and 6.5 kPa) during MVD on the structure of dried carrots was observed in scanning electron microscope (SEM) images. In both cases, the cell walls of the carrot tissue were damaged, which was associated with damage to the texture and a change in the shape and volume of the intercellular spaces. The dried materials obtained at a pressure of 3.5 kPa were characterized by a looser structure compared to the dried samples dried at a pressure of 6.5 kPa, the structure of which was more compact, and the free intercellular spaces were less visible. This can be explained by the different rates of water evaporation from the material, depending on the pressure, and consequently, the collapse of the internal structure of the tissue during drying [42]. Wang et al. [42] showed that during the vacuum blanching of carrots with water vapor, a rapid change in pressure from 20 kPa to atmospheric pressure at the level of 100 kPa causes the expansion of microchannels and, as a result, the cracking of cell walls. Therefore, carrot tissue becomes more susceptible to deformation. The multistage production process of dried carrots, including initial enrichment or osmotic dehydration, coating, convective drying, and final MVD, is a series of thermal operations that contribute to the intensive degradation of cell wall components and a decrease in sample elasticity [50]. As a result of the loss of water and turgor, cells shrink and lose their firmness. Cell walls may also be separated from the cytoplasm (plasmolysis) [41]. Wang et al. [23] and Xu et al. [50], discussing the cause of microstructure destruction of carrots, found that the thermal effect has the most significant influence, which degrades the structure of the cell membrane and the hemicellulose network of the cell wall and also reduces the solubility of pectin. These changes may reduce intercellular adhesion, consequently reducing hardness and gumminess.

Based on many microstructure photos of dried carrot, a more compact structure was found in samples after initial osmotic dehydration and with SA coating than after enrichment and pectin coating, especially of dried carrot produced from a 1.5% solution. However, the significant variation in cell size and their packing in carrot tissue may not justify these statements. Therefore, more detailed studies are needed on the state of water in dried carrot tissue. However, Karwacka et al. [51], in their studies, showed that a more significant addition of pectin at the level of 1.5% to freeze-dried multi-ingredient snacks increases the porous structure of these products, compared to the addition at the level of 0.5%.

#### 2.1.5. Sensory Evaluation of Selected Dried Carrot

**Figure 3:**
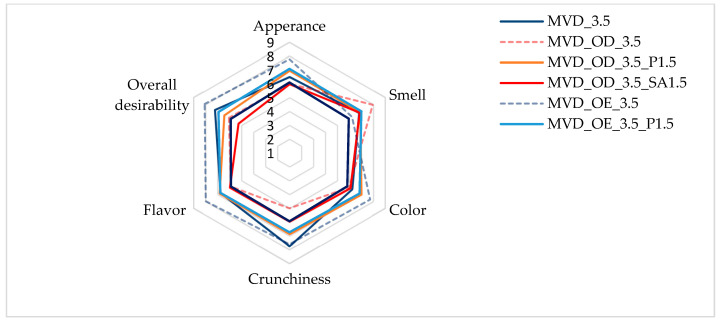
Osmotic treatment effect (enriching (OE)/dehydration (OD)) in chokeberry juice (NFC /concentrate) on sensory evaluation of dried carrots at a scale of 1–9. Code designations of dried samples are in Table S1.

As a summary of the obtained results, a sensory evaluation of selected dried carrot samples was performed. As shown in Figure 3, the evaluations of all the samples assessed on a 9-point scale were positive.

The highest scores, reaching 8 points in appearance, color, crunchiness, flavor, and overall desirability, were given to samples enriched in NFC chokeberry juice. Only the smell of these samples was rated lower, at about 6 points. Samples dehydrated osmotically in concentrated chokeberry juice obtained the highest score in terms of smell (about 8 points) and, at the same time, the lowest score for crunchiness (about 5 points). The less intense red color of the pre-enriched and dried carrot samples made the color of the samples more attractive compared to the osmotically dehydrated ones. According to Mohammadi et al. [52], in the sensory evaluation, dried products (carrots, broccoli, and oranges) using the radiant energy vacuum method led to improved taste and optical properties compared to freeze-drying, which is considered the most desirable. In the evaluation by Yusuf et al. [5], dried carrot samples of four colored varieties obtained by the combined osmotic dehydration (OD), convective drying (CD), and microwave–vacuum drying (MVD) methods turned out to be very attractive in sensorial and health-promoting properties. However, acceptance of individual sensory attributes varied depending on the carrot variety and the type of osmotic solution.

### 2.2. Influence of Osmotic Treatment, Edible Coating, and MVD Pressure on Chemical Properties of Dried Carrot

#### 2.2.1. Total Phenolic Content (TPC) of Fresh and MVD Carrot

**Figure 4:**
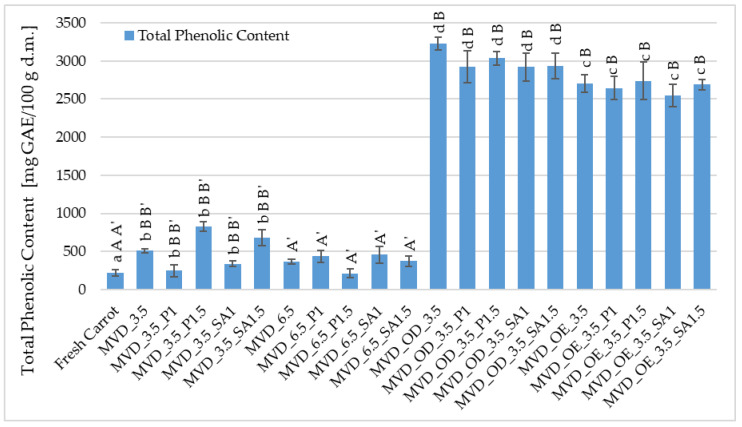
Total phenolic content (TPC) in dried carrots. Different letters mean homogeneous groups at *p* ≤ 0.05: a, b, c, d—osmotic treatment effect (enriching (OE)/dehydration (OD)) in NFC chokeberry juice/concentrate; A, B—coating type and concentration; and A’, B’—drying pressure. Code designations of dried samples are in Table S1.

The (TPC) in fresh carrots was about 255.8 mg GAE/100 g d.m., and after drying, it increased by 13-fold (Figure 4). In the previous series of studies by Ignaczak et al. [4], the TPC in fresh carrots was about 138 mg GAE/100 g d.m., and drying carrots caused a significant increase in TPC by 89.0%.

Differences in the total phenolic content in fresh carrots may result from the methodology used to determine it. According to Baryga et al. [53], these factors include the type of extraction solvent and its proportion to the sample mass, temperature, extraction time, and product storage conditions. These researchers demonstrated that in the case of beet pulp, using different solvents (80% methanol, 80% ethanol, 80% acetone, and water) resulted in very different TPC contents. When the TPC content after extraction with methanol and acetone did not differ significantly (366 and 381 mg CE/100 g d.m.), the TPC content after extraction with ethanol was three times lower, and with water, it was the highest (520 mg CE/100 g d.m.). They also noted that the results of tests with the Folin–Ciocalteu reagent may be overestimated because the reagent may react not only with polyphenols but also with amino acids and alkaloids, proteins, organic acids, polysaccharides, and vitamin C.

A significant effect of osmotic treatment was demonstrated, with osmotic dehydration (OD) having the most significant impact on increasing the TPC content in dried carrots, and the application of coatings after OD slightly reduced this increase in TPC. Such a significant increase in TPC in carrot samples resulted from the use of chokeberry juice concentrate in which the TPC content is high; according to Samborska et al. [54], in the concentrate of 65 °Bx, it was approximately 9871 mg GAE /100 g d.m. In strawberries dried using the microwave-convection and freeze-drying methods, osmotic pre-treatment in chokeberry juice concentrate with sucrose (1:1) resulted in an increase in the TPC content from 5.7 in the raw material to approximately 12.0 and 13.7 g GAE/100 g d.m. [55].

A significant effect of osmotic pre-treatment of carrots (OE/OD) and drying pressure was found on the total phenolic (TPC) content. In contrast, no effect of coatings on TPC was observed (Figure 4). In comparison to the raw material, the control samples dried at 3.5 and 6.5 kPa showed higher TPC values from 216 to 826 mg GAE/100 g d.m., with the higher pressure causing significantly lower TPC values, but mostly higher than in the raw material (216–458 mg GAE/100 g d.m.). In the osmotically dehydrated samples, the TPC content increased from 2919 in the coated samples to 3229 mg GAE/100 g d.m. in the uncoated samples and from 2543 to 2738 mg GAE/100 g d.m. in the enriched samples, with 2704 mg GAE/100 g d.m. in the uncoated OE samples (Figure 4). No effect of coatings on TPC content in dried carrot samples was observed, regardless of other factors.

Different conditions for drying half-slices of carrots, in terms of drying pressure, coatings, and especially osmotic treatment, allowed for a very beneficial increase in polyphenolic compounds in dried carrots. Compared to the control, in the case of osmotic enrichment (OE), the increase in TPC content was significant and, considering the coatings, on average, about 9% lower than with OD. These losses could be related to the leaching of native components from carrot tissue during coating application. Coatings prepared from 1.5% solutions provided a slightly better barrier against TPC loss than those from 1% coating solutions. However, these differences were not statistically significant. In the study by Mina et al. [39], pre-treatment with edible coatings (3% GA/ethanol) significantly improved the retention of phenolic compounds (47–55% TPC) in dried carrot slices compared to the control samples. They explained that the coating protected the samples from the oxidation of phenolic compounds. In previous studies by Ignaczak et al. [4], non-concentrated NFC juices were used. In this study, a concentrate of this juice was also used in addition to NFC chokeberry juice. Similarly to the previous research, drying caused a significant increase in TPC in enriched carrot samples. Regardless of the coatings used, concentrated chokeberry juice used for the osmotic dehydration of carrots resulted in significantly higher total phenolic content in dried carrots by 9–16% than in samples treated with NFC chokeberry juice. Compared to OE, the application of OD to carrot samples before drying resulted in a disproportionately smaller increase in TPC than the difference in the concentration of NFC juice and concentrated juice. It was expected that a higher concentration of concentrated chokeberry juice used for the osmotic dehydration of carrots would result in significantly more significant differences in TPC content. Therefore, these interesting observations should be investigated more thoroughly from the perspective of sustainable techniques and raw material savings in producing attractive carrot snacks. Consequently, it was planned to continue the research to assess the state of water in dried carrots concerning the penetration of enriching substances.

Other researchers have shown that the TPC content in dried carrot samples was significantly reduced, but some have indicated an increase compared to the content in the raw material. Amin et al. [45] found a decrease in the total phenolic content with increasing microwave power, from 189.5 in the raw material to 153.2 and 135.5 μg GAE/g in carrots dried at 170 and 340 W microwave power. They explained this by thermal degradation, oxidation, volatilization, and chemical transformations of phenolic compounds. These changes in atmospheric pressure conditions could occur quite intensively, in contrast to drying under reduced pressure, 3.5 and 6.5 kPa, where access to oxygen is limited, and the temperature at the exit from the drying chamber was not higher than 70 °C. According to de Mendonça et al. [46], pre-blanching also contributed to the increased retention of phenolic compounds, alongside the use of vacuum and low microwave power. At the same time, under MVD conditions, the lower boiling point of water has a beneficial effect on reducing the drying temperature of carrots. However, at higher MVD temperatures, uneven microwave heating and even local burns are relatively common [4]. Therefore, non-enzymatic Maillard reactions may also occur. Polyphenols can participate in these reactions, and different conformations can lead to the formation of new phenolic compounds, including at the expense of others, especially those with a higher molecular weight [56]. In the case of TPC, the concentration of carrots due to the removal of water, the reactions taking place, and various enrichment methods can increase their content. Also, Yusuf et al. [5] showed that preliminary OD of carrot in apple, chokeberry, and cherry juice concentrates increased the phenolic content in dried carrot samples of different varieties; TPC content was in the range of 442.52–508.63 mg/100 g product. Calín-Sánchez et al. [12] showed that, in addition to temperature and oxygen, the variability of phytochemical content may be influenced by the time of exposure to these conditions and light availability. The most crucial advantage of MVD over other methods is the significantly shortened drying time, which is essential in quickly achieving a moisture content of 15–20%, which limits the color changes of dried products caused by Maillard reactions [57], including the retention of phytochemicals. However, Béttega et al. [18] found that forming a porous structure induced by microwave–vacuum drying resulted in greater permeability to the emerging steam, influencing a more intensive degradation of some carrot components. Therefore, using coatings could reduce the losses of total phenolic content.

#### 2.2.2. Antioxidant Activity DPPH• and ABTS•+

**Figure 5:**
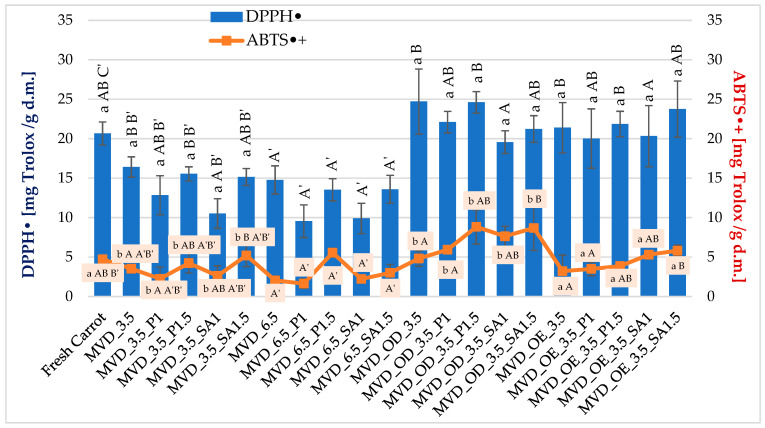
Antioxidant activity of dried carrots. Different letters mean homogeneous groups at *p* ≤ 0.05: a, b—osmotic treatment effect (enriching (OE)/dehydration (OD)) in NFC chokeberry juice/concentrate; A, B—coating type and concentration; and A’, B’, C’—drying pressure. Code designations of dried samples are in Table S1.

Antioxidant activity (AA) of fresh carrots determined by the DPPH• method was about 20.7 mg Trolox/g d.m., and by the ABTS•+ method, 4.7 mg Trolox/g d.m. (Figure 5).

In earlier studies by Ignaczak et al. [4], the antioxidant activity of fresh carrots measured by the DPPH• method was approximately 4.1 mg Trolox/g d.m., and by the ABTS•+ method, 13.3 mg Trolox/g d.m., and drying caused their increase to 7.5 and 52.0 Trolox/g d.m., respectively. Similarly, in the study by Keser et al. [44], AA in fresh carrots measured by the DPPH• method was lower and amounted to about 5.4 mg Trolox/g d.m. These differences could have been influenced by the properties of the raw material, growing conditions, including agrotechnical and environmental conditions (temperature and rainfall), maturity, time, storage conditions, and the determination methodology. These studies were carried out in autumn, and the previous ones in spring.

Generally, drying carrots caused a decrease in these indicators but an increase in the samples subjected to osmotic treatment. However, statistical analysis did not show a significant effect of osmotic treatment on the DPPH• value (Figure 5). The average values of this parameter ranged from 20.7 for fresh samples to 22.4 mg Trolox/g d.m. in those subjected to initial osmotic dehydration (OD). In the case of antioxidant activity measured using ABTS•+, a significantly higher antioxidant activity, by about 52%, was found in OD samples, compared to fresh carrot samples and those dried after OE enrichment. The effect of other factors on the DPPH• and ABTS•+ values was statistically significant. However, the relationships were varied (Figure 5). It was difficult to determine the effect of coatings on AA because, concerning AA in the raw material, significantly lower DPPH• values by about 19% were observed in samples with alginate coating (MVD_3.5_SA1), and higher by 1—6% in samples without coating and with pectin coating (MVD_3.5_P1.5).

Antioxidant activity measured by ABTS•+ in samples without coating and with pectin coating (MVD_3.5 and MVD_3.5_P1) was significantly lower by about 18%, but in samples with alginate coating (MVD_3.5_SA1.5), significantly higher by about 39%. AA of the remaining samples did not differ significantly from the activity of the raw material. However, the change in MVD pressure from 3.5 to 6.5 kPa in the case of both indicators led to a significant decrease in AA by about 25 and 40%, respectively. In earlier studies by Ignaczak et al. [4], drying caused an increase in DPPH• and ABTS•+ to 7.5 and 52.0 Trolox/g d.m., respectively. In the studies by Keser et al. [44], in comparison to the raw material (approx. 5.4 mM Trolox/g d.m.), AA measured by the DPPH• method in dried carrots decreased to 3.08–3.85 mM Trolox/g d.m., while in the studies by Amin et al. [45], in comparison to fresh carrots (85.2%), the percentage extinction of compounds with antioxidant properties decreased to 63.9%. They explained that this decrease was caused by high temperature, at which point the effective oxidation of antioxidant compounds could occur, reducing their antioxidant capacity. On the other hand, Özcan et al. [58] found that as a result of reduced moisture, antioxidant activity increased from 16.2% (control sample) to 92.3% in kiwi and from 11.4 to 90.6% in microwave-dried pepino fruits, but also in oven-dried (79.3–88.1%) ones, compared to fresh fruits. A significant increase in antioxidant activity also occurred in *Capparis spinosa* L. fruits, dried by microwave at 600 and 900 W, i.e., under high energy (temperature) [59].

The conducted studies showed AA differentiation depending on the method used. Active radicals are reduced in both methods under the influence of various antioxidant compounds, including various polyphenol compounds [60]. For example, flavonoids or carotenoids in carrots cause the reduction of ABTS•+ cation radicals, which may influence the overestimation of the obtained results of antioxidant activity. At the same time, it should be noted that dehydrated or enriched carrot samples showed higher antioxidant activity in most cases compared to fresh carrot samples. A significantly higher total phenolic content also characterized these samples. The coatings did not show an apparent effect on AA in the analyzed samples. However, it was observed that coatings produced from the 1.5% solution likely provided a greater amount of coating material on the samples, increasing the AA level, which could be a result of providing a greater protective barrier against factors such as oxygen or high temperature during drying. Some of the changes in the increased antioxidant activity of carrots can also be explained by the adjustment of MVD conditions and pre-treatments, including blanching [4], which, compared to other methods, results in a shorter drying time Wang et al. [25] and a higher concentration of nutrients and phytochemicals [61], which may limit thermal degradation and oxidative reactions.

### 2.3. Comprehensive Summary of Results

**Figure 6:**
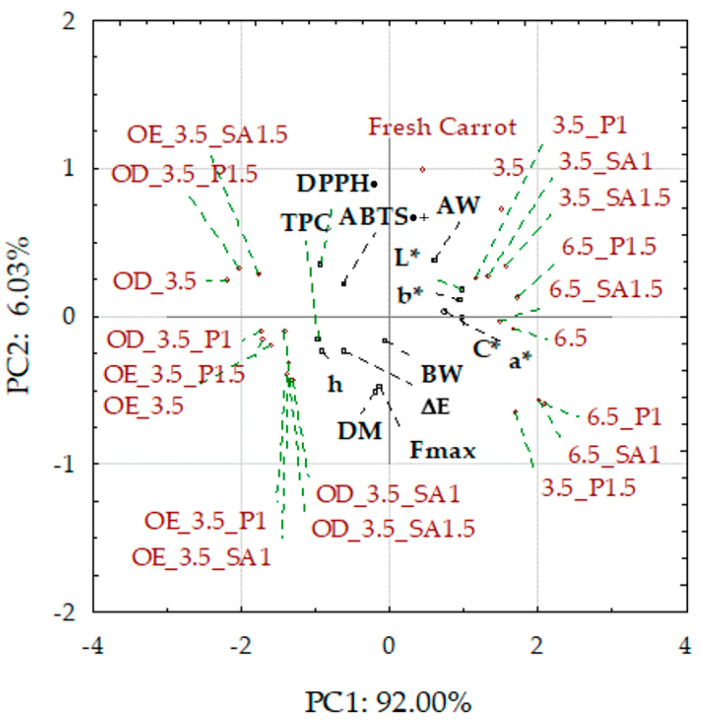
Biplot of PCA score plot and principal component loading plot PC1 and PC2. Explanations: L*, a*, b*, C*, h, and ΔE—color parameters and absolute color difference; AW—water activity; Fmax, BW—hardness and breaking work; DM—dry matter content; TPC—total phenolic content; and DPPH•/ABTS•+—antioxidant activity; code designations of dried samples are in Table S1.

Principal component analysis (PCA) was used to classify fresh and dried carrot samples concerning osmotic treatment, coating application, and drying pressure, depending on the values of the studied physicochemical parameters. Presented as a biplot (two graphs of the projection of variables on the factor plane), it allowed for the grouping of the fresh, control, and coated dried carrot samples, mainly on the right side of the surface, while the osmo-treated and dried samples were on the left side (Figure 6). Fresh carrots, control samples, and those with coatings from 1% solutions were characterized by lower dry matter content and higher water activity, especially those dried at a pressure of 6.5 kPa.

In addition, these samples showed higher values of color parameters L*, a*, b*, and C, when subjected to osmotic treatment, were characterized by significantly less vivid colors and significant darkening caused by the penetration of colored components of chokeberry juice, which also resulted in a significant increase in the content of phenolic compounds and antioxidant activity compared to the raw material. These samples showed different hardness and breaking work; however, the lowest hardness was characterized by the samples enriched with OE in NFC chokeberry juice. The graph shows that the antioxidant activity indices DPPH• and ABTS•+ and total phenolic content (TPC) are close. Therefore, the correlations of DPPH• and ABTS•+ with TPC are positive and significantly high, 0.85 and 0.65, respectively. Highly significant but negative correlations also apply to DPPH• and TPC with the color parameters of the samples after osmotic treatment, which is confirmed by the darkening of the color and the reduction of the share of yellow and red colors, as well as the intensity (vividness) of the color. Despite these significant changes, the color of the carrot samples was sensorily highly assessed, at 7–8 points on a 9-point scale. A high negative correlation of TPC with water activity (AW) was also found. This confirms the earlier observations that the reduction in water activity resulting from, among others, the increased dry matter content results in a higher share of phenolic compounds in dried carrots. Regarding texture, a significant negative correlation was found between hardness (maximum force in the compression test Fmax) and water activity and a positive correlation with DM. However, there was no correlation between the breaking work and these indicators. The hardness of dried carrots is mainly dependent on water activity. It is evident that increasing the AW of dried products is associated with a loss of hardness/crunchiness and leads to unacceptable gumminess for dry snacks [62].

## 3. Materials and Methods

### 3.1. Materials and Experimental Procedure

The carrots used for the study were from the “Tadeusz Karaś” horticultural farm, purchased in a large-format store in Warsaw. The roots had comparable dimensions (diameter of about 35 mm) and a similar intensity of the orange color. The carrots needed for both series of studies were stored in a cold store at a temperature of 2 ± 1 °C. The carrots were cut into 3 mm slices using a slicer and then cut into half-slices.

**Figure 7:**
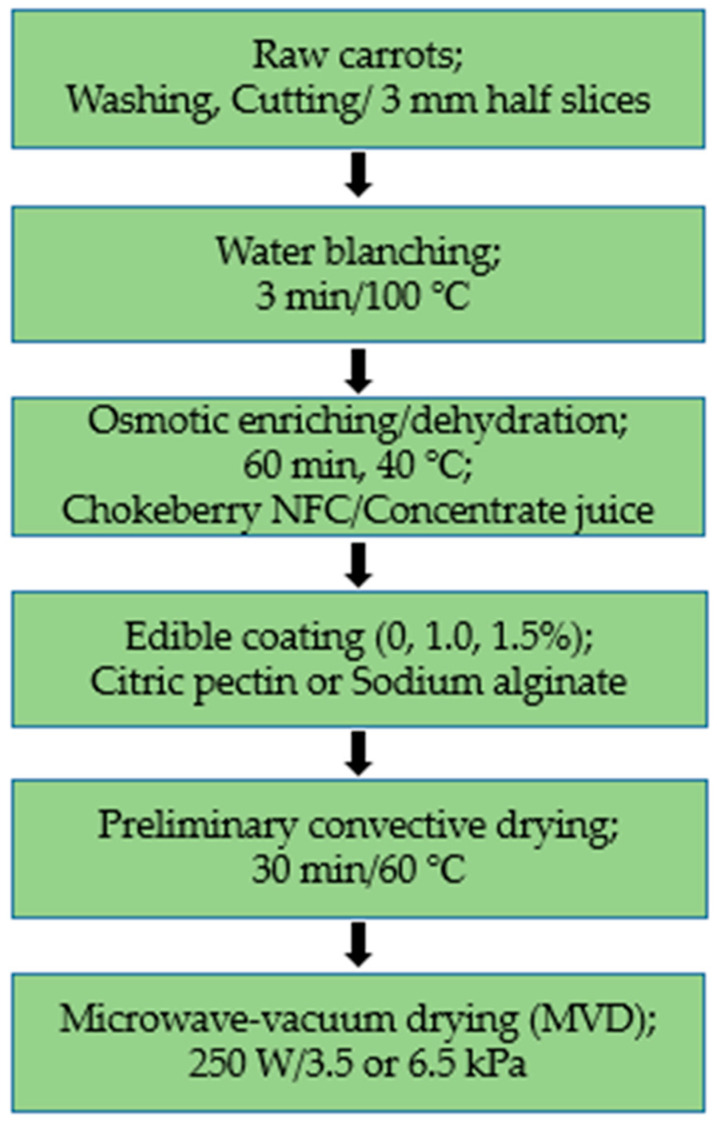
Scheme of the experimental procedure for drying carrots.

Additionally, as required for vegetables, carrot samples were subjected to pre-water blanching at 100 °C for 3 min and then cooled at room temperature. Figure 7 shows a schematic diagram of the experimental procedure for drying carrots.

### 3.2. Technological Methods

#### 3.2.1. Pre-Treatment Methods

##### Osmotic Enrichment/Osmotic Dehydration

Osmotic enrichment (OE) was performed using chokeberry juice NFC—not from concentrate (OLEOFARM sp. z o.o. Wrocław, Poland)—at a concentration of 19 ± 1 °Bx and osmotic dehydration (OD) in concentrated chokeberry juice (Gomar Pińczów, Poland) at a concentration of 67 ± 1 °Bx. The juice was stored at room temperature in dark glass bottles. Osmotic enrichment/dehydration was performed in a water bath (JW. Construction, Warsaw, Poland) at 40 °C for 60 min. After enrichment, the carrot half-slices were separated from the juice solution using a sieve and then dried on filter paper. The sample size was about 100 g. The sample weight to osmotic solution weight ratio was 1:2. Before and after the process, the samples were weighed on a technical scale (Type WPE 2000, RADWAG, Radom, Poland) with an accuracy of 0.1 g.

##### Edible Coatings Preparation and Application

The coating methodology consisted of preparing solutions of citrus pectin (P) and sodium alginate (SA) at concentrations of 1% and 1.5% (w/V), which were obtained by dissolving them in distilled water and stirring them using magnetic stirring (500 rpm) RCT basic IKAMAG stirrer (IKA-Werke GmnH & Co., Staufen im Breisgau, Germany). The film-forming solutions were heated at 50 °C for 15 min, and then glycerol (plasticizer) was added in an amount of 50% of the coating concentration. The sliced carrots were immersed in the film-forming solution for 1 min and then for about 5 s in a 2% (m/V) calcium lactate solution to enhance the coating effect. Then, the samples were placed on filter paper for pre-drying and then dried in a laboratory convection dryer (available at the Institute of Food Sciences of the Warsaw University of Life Sciences) at 60 °C for about 30 min.

#### 3.2.2. Microwave––Vacuum Drying (MVD)

Carrot samples were subjected to microwave–vacuum drying in a PROMIS-TECH dryer (Wrocław, Poland). The process was carried out at a pressure of 3.5 or 6.5 kPa. The microwave power was 250 W, and the maximum temperature of the vapors at the chamber outlet was 70 °C. Drying was carried out in 4 cycles, where cycles 1 and 3 used microwave action lasting 180–480 s, while cycles 2 and 4 used process stabilization lasting 210 s.

### 3.3. Physicochemical Determinations

#### 3.3.1. Dry Matter Content, Water Activity, and Color Parameters

Dry matter content was determined based on the difference in mass before and after drying the samples at 100 °C to constant mass for at least 4 h, according to the procedure described in the previous publication by Ignaczak et al. [4], similar to water activity and color parameters.

#### 3.3.2. Texture Analysis

The mechanical properties of dried carrots were tested using a TA-HD plus texturometer (Stable Micro Systems, Godalming, UK) based on a breaking test using a 20 mm support spacing. The head travel speed with a special tip was 1 mm/s. Single slices of dried carrots were tested, which were characterized by surface inhomogeneity caused by shrinkage and puffing. The measurement was performed in at least 10 repetitions. The maximum force obtained during the test (Fmax [N]) and the work mJ [N mm] were determined using the software included with the station, calculated as the product of half the area under the breaking curve and the head speed [20].

#### 3.3.3. Structure Analysis by Scanning Electron Microscopy (SEM)

Dried carrot samples of 5 × 5 mm in size were mounted in a metal holder and sputtered with a 5 μm layer of gold (Leica EM ACE200; Leica Mikrosysteme GmbH, Vienna, Austria). Then, the internal structure was photographed using a Phenom XL scanning electron microscope (Thermo Fisher Scientific, Waltham, MA, USA) at an accelerating voltage of 10 kV and a chamber pressure of 60 Pa. At least 5 images of each sample were taken at a magnification of 500× [63].

### 3.4. Sensory Evaluation

The sensory evaluation of 7 representative samples of dried carrots was conducted by a panel of 32 people aged 20–45, who were employees and students at the Warsaw University of Life Sciences. The study was conducted in stable temperature, humidity, and lighting conditions. The individual descriptors used to evaluate the dried carrot snacks were explained to the assessors so that they understood their meaning (qualitatively) and could scale their intensity (quantitatively). The following descriptors were defined: appearance, smell, color, crunchiness, flavor, and overall desirability. A 9-point hedonic scale was used (like extremely—9, like very much—8, like moderately—7, like slightly—6, neither like nor dislike—5, dislike slightly—4, dislike moderately—3, dislike very much—2 and dislike extremely—1). The carrot snacks were marked with codes and served on plastic trays. Fresh, non-carbonated water was provided to rinse the mouth.

### 3.5. Chemical Determinations

#### 3.5.1. Extract Preparation

Fresh and dried carrot samples were ground in an analytical mill (IKA A11 basic; IKA-Werke GmbH, Staufen, Germany). Extracts were prepared by extracting 0.3 g of material in 10 mL of 80% (*v*/*v*) ethanol aqueous solution. The process was carried out at room temperature 20 °C for 24 h on a Multi Reax shaker (Heidolph Instruments, Germany). The prepared extracts were centrifuged for 2 min at 3000 rpm using a MegaStar 600 laboratory centrifuge (VWR, Belgium). The extraction was duplicated for each sample [63]. The extracts prepared this way were used to determine the total phenolic content (TPC) and antioxidant activity (DPPH• and ABTS•+).

#### 3.5.2. Total Phenolic Content (TPC)

The total phenolic content (TPC) was determined spectrophotometrically using the Folin–Ciocalteu reagent [63]. A total of 10 μL of sample extract, 10 μL of distilled water, and 40 μL of fivefold diluted Folin–Ciocalteu reagent were added to 96-well plates and mixed. After 3 min, 250 μL of supersaturated sodium carbonate solution was added and left to incubate in the dark at 25 °C for 60 min. The blank was prepared by replacing the extract with the extraction reagent solution. The absorbance of the mixtures was measured at 750 nm using a Multiskan Sky plate reader (Thermo Electron Co., Waltham, Massachusetts, USA). The total phenolic content (TPC) was calculated using the calibration curve prepared for gallic acid, and the results were expressed as mg of gallic acid equivalents (GAE) per 100 g of dry matter. The determination was performed in triplicate for each extract.

#### 3.5.3. Antioxidant Activity (AA)

The antioxidant activity of dried carrot samples was determined using DPPH• and ABTS•+ radical solutions [63]. The working solutions were diluted with 80% (*v*/*v*) ethanol to obtain a solution with absorbance in the range of 0.680–0.720 at 515 nm and 734 nm for DPPH• and ABTS•+ radicals, respectively. A total of 10 µL of 10-fold diluted sample extract and 250 µL of radical working solution were added to a 96-well plate. The samples were incubated at 25 °C in the dark for 30 min for DPPH• radical samples and 6 min for ABTS•+ radicals. After this time, the absorbance of the solutions was measured using a Multiskan Sky plate reader (Thermo Electron Co., USA). Results are expressed in mg Trolox (TE) per g dry matter (d.m.). The analysis was performed in triplicate for each extract.

### 3.6. Statistical Analysis

Statistical analysis of data was performed in STATISTICA 13 PL. To demonstrate the effect of coatings on the tested quality indicators with at least 3 repetitions, a one-way analysis of variance and Tukey’s HSD test (post hoc) were used. To assess the relationship between indicators, Pearson correlation and principal component analysis (PCA) were performed. In addition, the biplot technique was used to investigate the similarity between data groups and samples, considering the multivariate approach.

## 4. Conclusions

The dried carrot samples proved to be suitably preserved, and the selection of pre-treatment and microwave–vacuum drying (MVD) conditions allowed the obtaining carrot snacks with attractive sensory properties in terms of color, smell, and texture, and due to the enrichment in chokeberry juice/concentrate, with beneficial health-promoting properties. Pre-enrichment of carrots in chokeberry juice NFC increased dry matter content (DM) more than osmotic dehydration in chokeberry juice concentrate and untreated samples. Higher MVD pressure in the 3.5–6.5 kPa range caused an unfavorable decrease in dry matter content and increased water activity.

Concerning the assumed research objective, using edible coatings did not bring the expected result regarding the control samples and those subjected to osmotic treatment (enrichment/dehydration) in chokeberry juice/concentrate. Coatings did not affect the total phenolic content, and the antioxidant activity in dried carrots was varied, lower or higher than in the raw material. However, the coatings were useful in shaping the sensory quality, especially in the color and texture.

The coatings contributed to a more favorable color change than in the raw material through greater lightness, red and yellow colors, and color saturation, but a decrease in the color tone, manifested by a transition from yellow to orange-red. Conversely, the color saturation decreased significantly in the osmotic-treated samples, increasing the color hue. Obtaining the desired crispy and crunchy texture of dried carrots requires an appropriate selection of MVD conditions, taking into account osmotic treatment and edible coatings. In the sensory evaluation, the crunchiness of the control samples and those enriched with pectin coating was the highest, at about 8 on a 9-point scale. All seven selected samples were assessed positively; the lowest score was about 5.0. A significant negative correlation was found between the grinding work and dry matter content of dried carrots, but no correlation was found between water activity and texture indices. Higher dry matter content reduced the value of the breaking work, which was associated with a more delicate, crunchy structure of dried carrots.

## Figures and Tables

**Table 1:** Dry matter content and water activity in dried carrot; coding markings of MVD samples (Table S1): 3.5 or 6.5—drying pressure, OD—osmotic dehydration in concentrated chokeberry juice, OE—osmotic enrichment in chokeberry juice NFC, P1 or P1.5—pectin film-forming solution with a concentration of 1 or 1.5%, SA1 or SA1.5—sodium alginate film-forming solution with a concentration of 1 or 1.5%.

	Type of Sample	Dry Matter Content, DM [%]		Water Activity, AW [-]	
1.	Fresh Carrot	13.51 ± 0.48		0.983 ± 0.00	
2.	MVD_3.5	95.95 ± 0.33		0.427 ± 0.01	
3.	MVD_3.5_P1	91.49 ± 0.29		0.424 ± 0.00	
4.	MVD_3.5_P1.5	95.57 ± 0.93		0.334 ± 0.00	
5.	MVD_3.5_SA1	91.68 ± 1.41		0.416 ± 0.00	
6.	MVD_3.5_SA1.5	96.05 ± 0.28		0.332 ± 0.01	
7.	MVD_6.5	93.44 ± 0.42		0.456 ± 0.01	
8.	MVD_6.5_P1	93.58 ± 0.07		0.430 ± 0.00	
9.	MVD_6.5_P1.5	88.30 ± 0.63		0.503 ± 0.01	
10.	MVD_6.5_SA1	84.60 ± 0.67		0.578 ± 0.00	
11.	MVD_6.5_SA1.5	85.26 ± 0.03		0.589 ± 0.00	
12.	MVD_OD_3.5	95.08 ± 0.98		0.277 ± 0.01	
13.	MVD_OD_3.5_P1	96.58 ± 0.93		0.258 ± 0.00	
14.	MVD_OD_3.5_P1.5	97.99 ± 0.02		0.164 ± 0.00	
15.	MVD_OD_3.5_SA1	96.44 ± 1.34		0.285 ± 0.00	
16.	MVD_OD_3.5_SA1.5	94.71 ± 0.89		0.269 ± 0.02	
17.	MVD_OE_3.5	99.52 ± 0.14		0.235 ± 0.02	
18.	MVD_OE_3.5_P1	98.31 ± 0.36		0.239 ± 0.02	
19.	MVD_OE_3.5_P1.5	99.08 ± 0.18		0.237 ± 0.00	
20.	MVD_OE_3.5_SA1	99.31 ± 0.37		0.266 ± 0.00	
21.	MVD_OE_3.5_SA1.5	97.22 ± 0.93		0.260 ± 0.00	
**One-factor analysis of variance** (*—significance effect when *p* ≤ 0.05)					
**Factors**		**P-probability/Homogenous groups**			
Type of osmotic treatment (a, b, c); analyzed samples: 2–6, 12–21		0.0001 *	No ^a^ OD ^b^ OE ^c^	0.0000 *	No ^b^ OD ^a^ OE ^a^
Coating type and concentration (A); analyzed samples: 2–6, 12–21		0.6122	No ^A^ SA1 ^A^ SA1.5 ^A^ P1 ^A^ P1.5 ^A^	0.4346	No ^A^ SA1 ^A^ SA1.5 ^A^ P1 ^A^ P1.5 ^A^
Drying pressure (A’, B’); analyzed samples: 2–11		0.0028 *	3.5 ^A’^ 6.5 ^B’^	0.0001 *	3.5 ^A’^ 6.5 ^B’^
**Correlation** (**—significance effect when *p* ≤ 0.05)					
Dry Matter/Water Activity −0.9050 **					

Explanations: No—no treatment (control); OE—osmotic enriching; OD—osmotic dehydration.

**Table 2:** Color parameters of dried carrot; coding markings of dried samples are in Table S1.

	Type of Sample	Parameter L*		Parameter a*		Parameter b*		ΔE	
1.	Fresh Carrot	40.24 ± 0.09		15.28 ± 0.12		26.53 ± 0.21		-	
2.	MVD_3.5	59.32 ± 4.47		12.74 ± 2.06		32.46 ± 3.85		20.45 ± 4.85	
3.	MVD_3.5_P1	51.14 ± 0.91		34.16 ± 5.72		46.22 ± 2.17		29.73 ± 3.49	
4.	MVD_3.5_P1.5	44.13 ± 4.49		15.43 ± 2.82		32.06 ± 2.48		7.96 ± 3.49	
5.	MVD_3.5_SA1	52.43 ± 4.19		22.92 ± 3.08		48.78 ± 3.26		26.69 ± 5.01	
6.	MVD_3.5_SA1.5	53.99 ± 2.77		14.23 ± 1.91		37.45 ± 1.87		17.78 ± 2.50	
7.	MVD_6.5	49.17 ± 2.83		6.87 ± 1.51		20.13 ± 1.07		14.03 ± 2.17	
8.	MVD_6.5_P1	52.98 ± 8.45		19.91 ± 2.21		37.06 ± 4.55		17.60 ± 8.85	
9.	MVD_6.5_P1.5	52.07 ± 1.57		36.94 ± 2.54		42.13 ± 0.94		29.26 ± 2.27	
10.	MVD_6.5_SA1	51.97 ± 3.12		27.54 ± 6.29		49.28 ± 3.42		28.92 ± 4.74	
11.	MVD_6.5_SA1.5	47.99 ± 0.99		28.48 ± 4.41		42.50 ± 3.30		22.38 ± 4.13	
12.	MVD_OD_3.5	20.25 ± 1.67		8.44 ± 1.69		2.18 ± 1.03		32.26 ± 2.06	
13.	MVD_OD_3.5_P1	20.88 ± 1.82		7.32 ± 0.77		1.72 ± 0.39		32.48 ± 1.44	
14.	MVD_OD_3.5_P1.5	21.84 ± 1.43		3.65 ± 1.02		2.11 ± 0.67		32.74 ± 1.24	
15.	MVD_OD_3.5_SA1	24.19 ± 2.67		6.69 ± 1.61		4.87 ± 1.43		28.35 ± 2.74	
16.	MVD_OD_3.5_SA1.5	26.26 ± 2.75		8.03 ± 1.70		5.60 ± 2.09		26.24 ± 3.42	
17.	MVD_OE_3.5	19.26 ± 1.80		8.24 ± 0.89		1.54 ± 0.38		33.42 ± 0.97	
18.	MVD_OE_3.5_P1	19.81 ± 1.47		8.29 ± 1.19		1.46 ± 0.65		33.11 ± 1.56	
19.	MVD_OE_3.5_P1.5	18.12 ± 2.20		3.92 ± 0.76		0.84 ± 0.28		35.78 ±1.71	
20.	MVD_OE_3.5_SA1	19.99 ± 1.63		6.23 ± 1.44		0.79 ± 0.26		34.02 ± 1.18	
21.	MVD_OE_3.5_SA1.5	21.87 ± 2.55		3.89 ± 1.15		1.42 ± 0.19		33.19 ± 1.72	
**One-factor analysis of variance** (*—significance effect when *p* ≤ 0.05)									
**Factors**		**P-probability/ Homogenous groups**							
Type of osmotic treatment (a, b, c, d); analyzed samples: 1–6, 12–21		0.0000 *	Fresh ^c^ No ^d^ OD ^b^ OE ^a^	0.0000 *	Fresh ^b^ No ^b^ OD ^a^ OE ^a^	0.0000 *	Fresh ^b^ No ^c^ OD ^a^ OE ^a^	0.0000 *	- No ^a^ OD ^b^ OE ^c^
Coating type and concentration (A, B, C); analyzed samples: 1–6, 12–21		0.0021 *	Fresh ^C^ No ^B^ SA1 ^B^ SA1.5 ^B^ P1 ^AB^ P1.5 ^A^	0.0001 *	Fresh ^BC^ No ^AB^ SA1 ^AB^ SA1.5 ^A^ P1 ^C^ P1.5 ^A^	0.0000 *	Fresh ^C^ No ^A^ SA1 ^B^ SA1.5 ^AB^ P1 ^B^ P1.5 ^A^	0.0121 *	- No ^AB^ SA1 ^AB^ SA1.5 ^A^ P1 ^AB^ P1.5 ^B^
Drying pressure (A’, B’); analyzed samples: 2–11		0.0001 *	Fresh ^A’^ 3.5 ^B’^ 6.5 ^B’^	0.1137	Fresh ^A’^ 3.5 ^A’^ 6.5 ^A’^	0.0148 *	Fresh ^A’^ 3.5 ^B’^ 6.5 ^B’^	0.4094	- 3.5 ^A’^ 6.5 ^A’^
**Correlation** (**—significance effect when *p* < 0.05)									
		L*/a*		L*/b*	a*/b*	L*/ΔE		a*/ΔE	b*/ΔE
		0.7325 **		0.9403 **	0.8777	−0.6800		−0.2142	−0.5278

**Table 3:** Selected photos of the general appearance (section A) and SEM imaging of the internal structure at magnification 200× (section B), and 500× (section C) of microwave–vacuum dried carrot with osmotic treatment effect (enriching (OE)/dehydration (OD)) in NFC chokeberry juice/concentrate, coating type, and concentration and drying pressure. Code designations of dried samples are in Table S1.

Sample	A	B	C
MVD_3.5	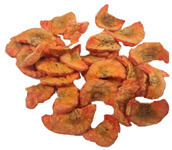	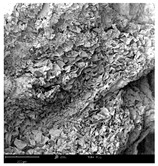	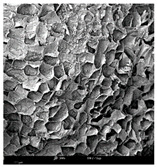
MVD_6.5	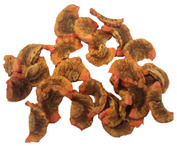	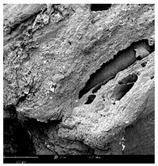	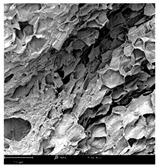
MVD_3.5_P1.5	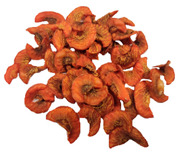	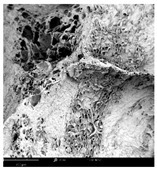	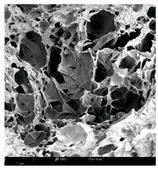
MVD_6.5_SA1.5	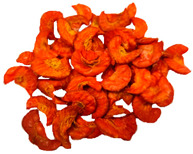	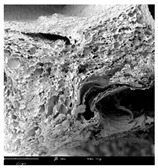	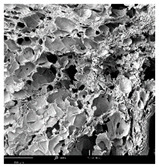
MVD_OD_3.5_SA1	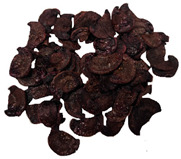	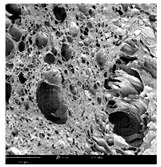	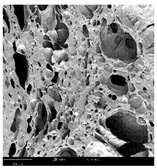
MVD_OE_3.5_P1	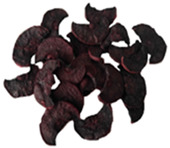	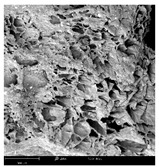	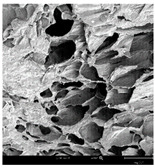

## Data Availability

The data presented in this study are available on request from the corresponding author.

## Supplementary Materials

The following supporting information can be downloaded at https://www.mdpi.com/article/10.3390/molecules30091877/s1, Table S1. List of abbreviations (codes) of different carrot samples and pre-treatment, Table S2. Photos of overall appearance (section A) and SEM imaging of the internal structure at magnification 200× (section B) and 500× (section C) of microwave–vacuum-dried carrot with osmotic treatment effect (enriching (OE)/dehydration (OD)) in NFC chokeberry juice/concentrate, coating type, and concentration and drying pressure.
