# Supplementary material for: The Influence of Osmotic Treatment, Edible Coatings Application, and Reduced Pressure on Microwave–Vacuum-Dried Carrot Properties
Source: Molecules. 2025 Apr 23;30(9):1877. doi: 10.3390/molecules30091877 (PMC12073827; doi:10.3390/molecules30091877)
Supplement: Supplementary file 1 [file molecules-30-01877-s001.zip › molecules-3543375-supplementary.pdf]

Supplementary Materials:

The Influence of Edible Coatings Application, Osmotic Treatment, and Reduced Pressure on Microwave–Vacuum Dried Carrot Properties

Table S1. List of abbreviations (codes) of different carrot samples and pre-treatment.

Abbreviations	Drying Pressure [kPa]	Osmotic Treatment	Edible Coating Type and Concentration
MVD_3.5	3.5	No treatment	No coating
MVD_3.5_P1	3.5	No treatment	1% Pectin
MVD_3.5_P1.5	3.5	No treatment	1.5% Pectin
MVD_3.5_SA1	3.5	No treatment	1% Sodium Alginate
MVD_3.5_SA1.5	3.5	No treatment	1.5% Sodium Alginate
MVD_6.5	6.5	No treatment	No coating
MVD_6.5_P1	6.5	No treatment	1% Pectin
MVD_6.5_P1.5	6.5	No treatment	1.5% Pectin
MVD_6.5_SA1	6.5	No treatment	1% Sodium Alginate
MVD_6.5_SA1.5	6.5	No treatment	1.5% Sodium Alginate
MVD_OD_3.5	3.5	Osmotic Dehydration	No coating
MVD_OD_3.5_P1	3.5	Osmotic Dehydration	1% Pectin
MVD_OD_3.5_P1.5	3.5	Osmotic Dehydration	1.5% Pectin
MVD_OD_3.5_SA1	3.5	Osmotic Dehydration	1% Sodium Alginate
MVD_OD_3.5_SA1.5	3.5	Osmotic Dehydration	1.5% Sodium Alginate
MVD_OE_3.5	3.5	Osmotic Enrichment	No coating
MVD_OE_3.5_P1	3.5	Osmotic Enrichment	1% Pectin
MVD_OE_3.5_P1.5	3.5	Osmotic Enrichment	1.5% Pectin
MVD_OE_3.5_SA1	3.5	Osmotic Enrichment	1% Sodium Alginate
MVD_OE_3.5_SA1.5	3.5	Osmotic Enrichment	1.5% Sodium Alginate

Table S2. Photos of overall appearance (section A) and SEM imaging of the internal structure at magnification 200x (section B), and 500x (section C) of microwave-vacuum dried carrot with osmotic treatment effect (enriching (OE)/dehydration (OD)) in NFC chokeberry juice/concentrate, coating type and concentration and drying pressure.

Sample	A	B	C
MVD_3.5	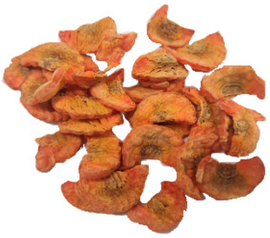	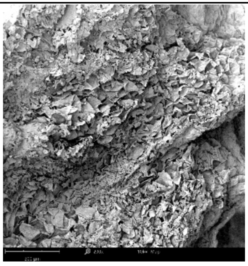	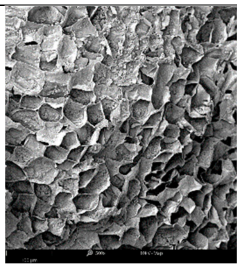
MVD_6.5	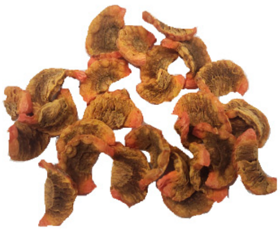	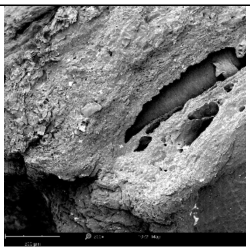	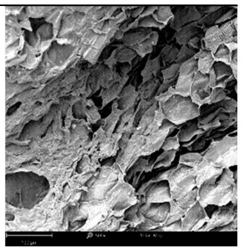

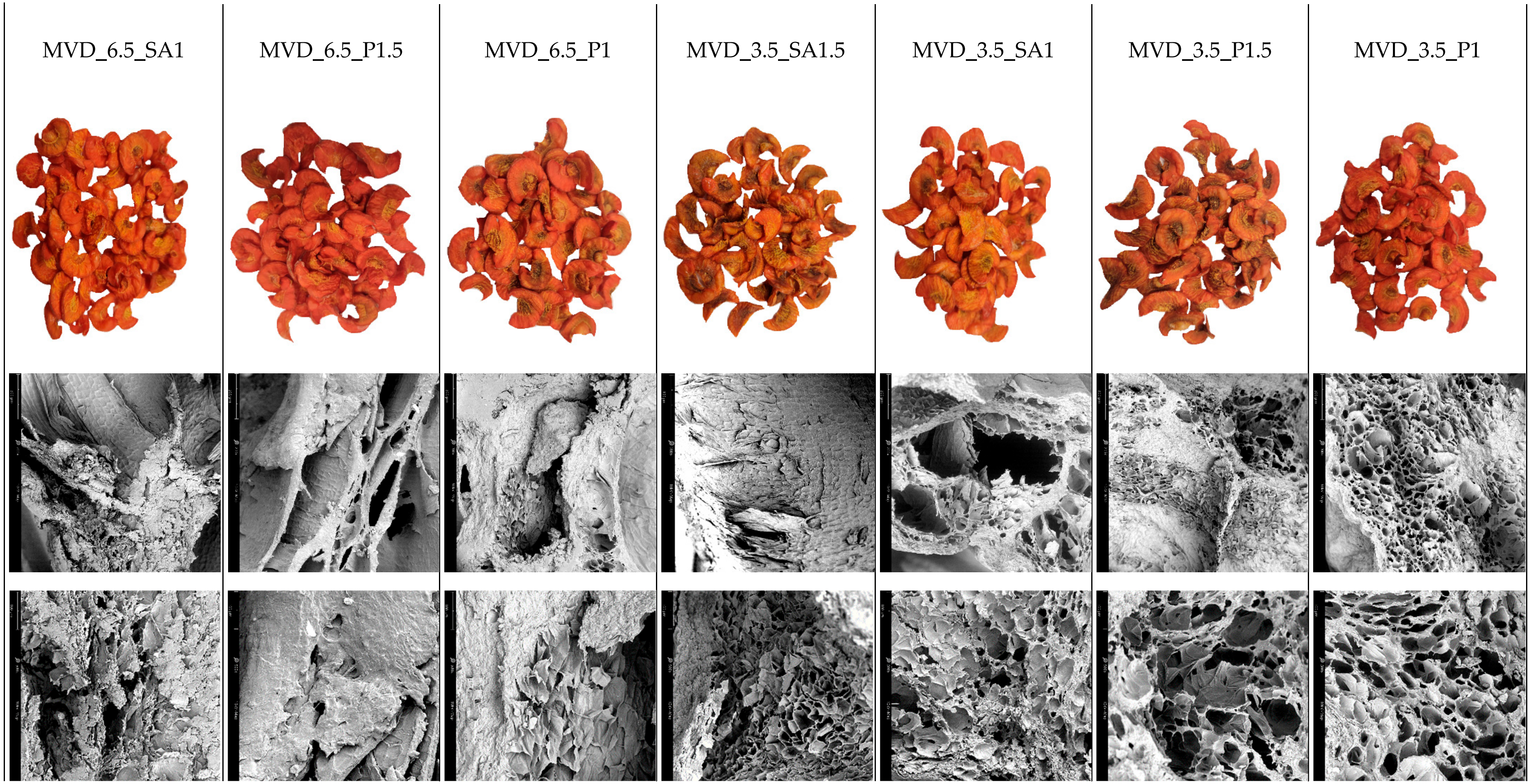
MVD_6.5_SA1
MVD_6.5_P1.5
MVD_6.5_P1
MVD_3.5_SA1.5
MVD_3.5_SA1
MVD_3.5_P1.5
MVD_3.5_P1

MVD_6.5_SA1.5	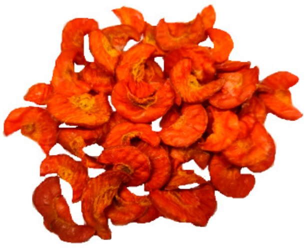	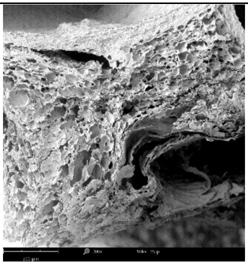	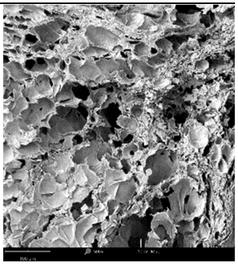
MVD_OD_3.5	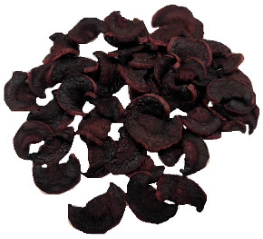	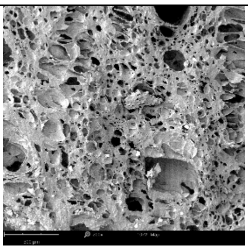	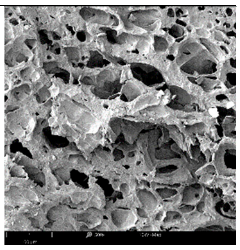
MVD_OD_3.5_P1	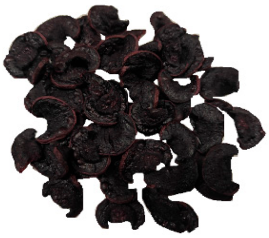	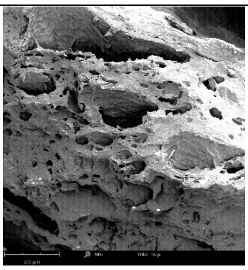	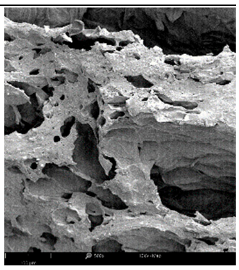
MVD_OD_3.5_P1.5	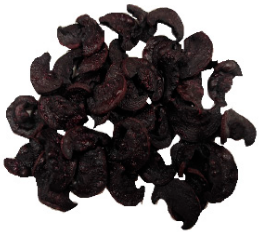	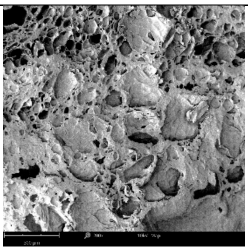	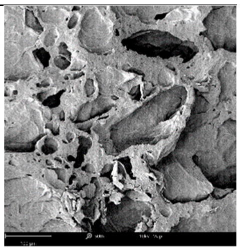
MVD_OD_3.5_SA1	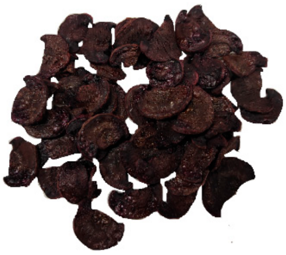	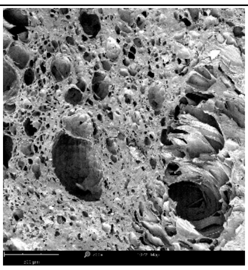	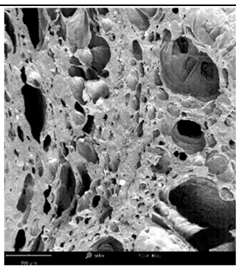
MVD_OD_3.5_SA1.5	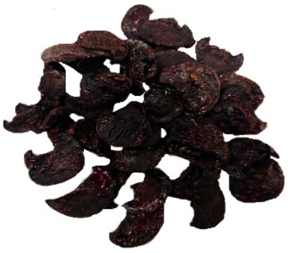	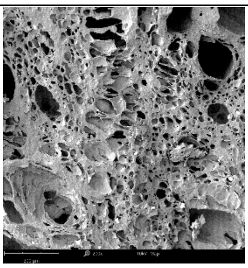	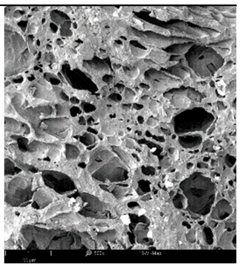
MVD_OE_3.5	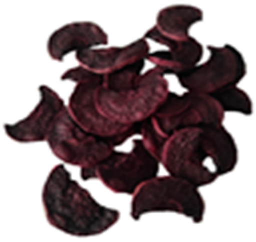	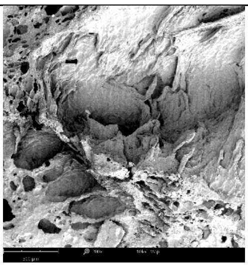	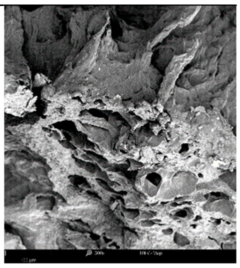

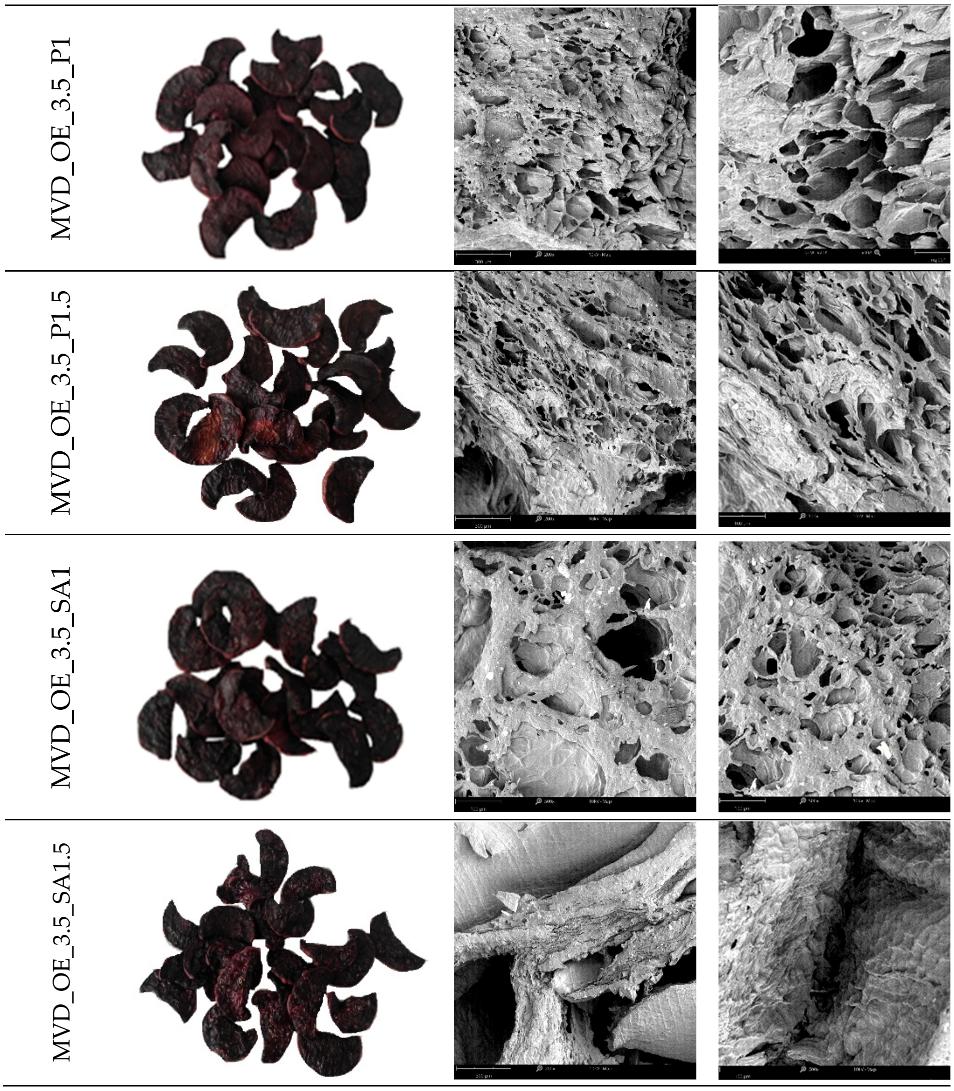
MVD_OE_3.5_P1
MVD_OE_3.5_P1.5
MVD_OE_3.5_SA1
MVD_OE_3.5_SA1.5
